# Prevalence of Bacteria and Intestinal Parasites among Food-handlers in Gondar Town, Northwest Ethiopia

**DOI:** 10.3329/jhpn.v26i4.1887

**Published:** 2008-12

**Authors:** Gashaw Andargie, Afework Kassu, Feleke Moges, Moges Tiruneh, Kahsay Huruy

**Affiliations:** 1 Department of Health Management and Health Economics, School of Public Health; 2 Department of Microbiology and Parasitology; 3 Department of Medical Laboratory, Gondar College of Medicine and Health Sciences, University of Gondar, PO Box 196, Gondar, Ethiopia

**Keywords:** Bacteria, Cross-sectional studies, Diarrhoeal diseases: Food-handlers, Hygiene, Intestinal diseases, Parasitic, Parasites, Ethiopia

## Abstract

Food-handlers with poor personal hygiene working in food-service establishments could be potential sources of infection due to pathogenic organisms. The study was undertaken to determine the prevalence of bacteria and intestinal parasites among 127 food-handlers working in the cafeterias of the University of Gondar and the Gondar Teachers Training College, Gondar, Ethiopia. Fingernail contents of both the hands and stool specimens were collected from all the 127 food-handlers. The samples were examined for bacteria and intestinal parasites following standard procedures. Coagulase-negative staphylococci were the predominant bacteria species (41.7%) isolated from fingernail contents, followed by *Staphylococcus aureus* (16.5%), *Klebsiella* species (5.5%), *Escherichia coli* (3.1%), *Serratia* species (1.58%), *Citrobacter* species (0.8%), and *Enterobacter* species (0.8%). *Shigella* species were isolated from stool samples of four food-handlers (3.1%). None of the food-handlers was positive for *Salmonella* species and *Shigella* species in res-pect of their fingernail contents. No intestinal parasites were detected from fingernail contents. Intestinal parasites detected in the stools of the food-handlers included *Ascaris lumbricoides* (18.11%), *Strongyloides stercoralis* (5.5%), *Entamoeba histolytica/dispar* (1.6%), *Trichuris trichiura* (1.6%), hookworm species (0.8%), *Gardia lamblia* (0.8%), and *Schistosoma mansoni* (0.8%); 1.6% of the study subjects were positive for each of *A. lumbricoides, T. trichiura*, hookworm, and *G. lamblia*. The findings emphasize the importance of food-handlers as potential sources of infections and suggest health institutions for appropriate hygienic and sanitary control measures.

## INTRODUCTION

Diarrhoeal diseases, mostly caused by foodborne or waterborne microbial pathogens, are leading causes of illness and deaths in developing countries, killing an estimated 1.9 million people annually at the global level. Even in developed countries, an estimated one-third of the population are affected by microbiological foodborne diseases each year (1). In contrast, Kaferstein and Abdussalam reported that up to 10% of the population of industrialized countries might suffer annually from foodborne diseases (2).

However, it can be expected that a large number of illnesses remain under-reported as only the most serious cases are usually investigated. On the other hand, many foodborne illnesses share common symptoms and cannot be distinguished by the symptoms alone. Diagnosis of a foodborne illness can only be made after considering the recent food-consumption history of a patient and performing proper laboratory tests for disease-producing parasites, bacteria, and bacterial toxins (3). In addition, health departments may not detect food-borne illness for several reasons. Merely, a small proportion of people infected with enteric pathogens seeks treatment and consequently submits specimens for testing. Even a smaller proportion of people tests positive for a pathogen that healthcare providers and laboratories notify to the health department (4).

Transmission of intestinal parasites and enteropathogenic bacteria is affected directly or indirectly through objects contaminated with faeces. These include food, water, nails, and fingers, indicating the importance of faecal-oral human-to-human transmission (5). Accordingly, food-handlers with poor personal hygiene working in food-serving establishments could be potential sources of infections of many intestinal helminths, protozoa, and enteropathogenic bacteria (6). Food-handlers who harbour and excrete intestinal parasites and enteropathogenic bacteria may contaminate foods from their faeces via their fingers, then to food processing, and finally to healthy individuals (2). Compared to other parts of the hand, the area beneath fingernails harbours the most microorganisms and is most difficult to clean (7). A sudden outbreak of food poisoning due to *Salmonella* Newport occurred from 31 December 1991 to 4 January 1992 among 344 students, and 23% of them manifested disease in the study area (University of Gondar), Gondar, Ethiopia (8). Although there was a sudden outbreak long time ago in the University of Gondar, no study was conducted in that area. Therefore, this study was aimed at assessing the prevalence of bacteria and intestinal parasites in food-handlers of Gondar town, Northwest Ethiopia.

## MATERIALS AND METHODS

A cross-sectional study was conducted among 127 food-handlers working in the cafeterias of the University of Gondar and in the Gondar Teachers Training College during 2-27 February 2003. The cafeterias served for 1,553 students, of which 67.6% were from the University of Gondar, and the remaining students were from the Gondar Teachers Training College. These cafeterias were selected for the study because mass provisions of food service cause a potential source of transmitting infections.

All the food-handlers (47 males and 80 females) working in the cafeterias of the institutes who did not take treatment for any intestinal ailment within the three months prior to the study were included. Seventy-four food-handlers were from the University of Gondar, and the remaining food-handlers (n=53) were from the Gondar Teachers Training College.

A structured questionnaire was used for collecting information on age, sex, educational level, and hygienic status of each food-handler. Samples of fingernail contents were collected from both the hands of each subject using sterile-moistened cotton-tipped swab and placed into a sterile test tube. Stool sample was collected from each subject in a clean stool cup.

All the samples were cultured into the plates of *Salmonella*-*Shigella* agar (Oxoid), MacConkey agar (Difco), and Blood agar (Oxoid). After incubation for 24 hours at 37 oC, the plates were examined, and bacterial species were identified following standard procedures (9). The fingernail contents and stool samples were also examined microscopically for intestinal parasites following direct wet mount preparations in normal saline and iodine solution (10). The formalin ether concentration sedimentation procedure was also followed to check for intestinal parasites in the stool samples (10). Data were entered and analyzed using the Epi Info (version 2000) statistical packages.

### Ethical clearance

Ethical clearance was obtained from the Research and Publication Office of the University of Gondar, and the objective of the study was explained to the study participants to get informed verbal consent.

## RESULTS

The study included all the 127 food-handlers, and the response rate was 100%. The large majority (72.4%) of the food-handlers were young adults aged 20-40 years. Only 58.3% of the food-handlers had education above elementary school level. Eleven percent of the food-handlers did not practise hand-washing after visiting toilet (Table 1).

**Table 1.** Sociodemographic characteristics of food-handlers in students’ cafeterias of two higher institutions in Gondar

Characteristics	Frequency	%
Age (years)		
20-40	92	72.4
>40	35	27.6
Sex		
Male	47	37.0
Female	80	63.0
Educational status		
Illiterate	3	2.4
Can read and write	5	3.9
1-6 grade	45	35.4
7-10 grade	47	37.0
11-12 grade	27	21.3
Years of service		
1-5	58	45.7
6-10	8	6.3
11-20	35	51.2
>20	26	20.5
Hand-washing practice after toilet		
Yes	113	89.0
No	14	11.0

Table 2 shows bacterial species isolated from cultures of fingernail contents. Of the 127 samples, cultures of fingernail contents were found to be positive for coagulase-negative staphylococci (41.7%), *S. aureus* (16.5%), *Klebsiella* species (5.5%), *Escherichia coli* (3.1%), *Serratia* species (1.6%), *Citrobacter* species (0.8%), and *Enterobacter* species (0.8%). However, no intestinal parasites were detected from the samples of fingernail contents.

**Tables 2.** Frequency and type of bacteria isolated from fingernail contents in food-handlers working in students’ cafeterias of two higher institutions in Gondar

Bacteria isolated	Frequency	%
Coagulase-negative staphylococci	53	41.7
*S. aureus*	21	16.5
*Klebsiella* species	7	5.5
*E. coli*	4	3.1
*Serratia* species	2	1.6
*Citrobacter* species	1	0.8
*Enterobacter* species	1	0.8

Stool cultures revealed the isolation of *Shigella* species from four (3.1%) food-handlers. No other enteropathogenic bacteria were isolated from the stools.

Direct microscopic and concentration techniques were used for identifying intestinal parasites from the 127 stool specimens. Thirty-seven (29.1%) stool specimens were positive for different intestinal parasites. *A. lumbricoides* was the most prevalent parasite (18.1%), followed by *S. stercoralis* (5.5%) (Table 3); 1.6% of the study subjects were positive for each of *A. lumbricoides, T. trichiura*, hookworm, and *G. lamblia*.

**Table 3.** Type and prevalence of intestinal parasites in stool samples of food-handlers in students’ cafeterias of two higher institutions in Gondar

Parasite species isolated	Frequency	%
*A. lumbricoides* and T. trichiura*	23	18.1
*S. stercoralis*	7	5.5
*E. histolytica/dispar*	2	1.6
*T. trichiura*	2	1.6
*G. lamblia*	1	0.8
Hookworm and *G. lamblia*	1	0.8
*S. mansoni*	1	0.8
Total	37	29.1

*22 study subjects had *A. lumbricoides*, but only one individual was infected by both *A. lumbricoides* and *T. trichiura*

## DISCUSSION

In this study, 29.1% and 3.1% of the 127 subjects were positive for stool parasites and enteropathogenic bacterial species respectively. In addition, other species of bacteria were also isolated from their fingernail contents. These indicate the health status and very poor hygiene practices of the food-handlers working in the cafeterias.

Several authors from allover the world have stressed the importance of food-handlers as threats in the transmissions of parasitic and bacterial diseases (11-20). In fact, a prerequisite for the control and prevention of intestinal parasitosis and diseases due to enteropathogenic bacteria are a clear understanding of their epidemiology as the information guides in the design of most practical and economic control and prevention measures.

The high (29.1%) prevalence of intestinal parasites in the stools of the food-handlers in our study was in agreement with the findings of other studies in the region and other parts of the country (21), indicating a high prevalence of soil-transmitted helminths and intestinal protozoans. Such a high prevalence of intestinal parasites is largely due to poor personal hygiene practices and environmental sanitation, lack of supply of safe water, poverty, ignorance of health-promotion practices, and impoverished health services.

Stool cultures of four food-handlers were positive for *Shigella* species. This may indicate their low educational status and may lead to outbreaks of bacillary dysentery among the student population. Interestingly, *Shigella* species were isolated from trays which were prepared and ready for use. This may also be a potential source of contamination and outbreaks of disease due to *Shigella*. The hygiene situation of the food-handlers was further challenged by the isolation of several species of bacteria, including *S. aureus*, and intestinal bacterial species, such as *Klebsiella, Enterobacter*, and *Citrobacter. E. coli and Serratia* species were isolated from their fingernail contents, supporting the notion of contamination by faecal bacteria due to inadequate hand-washing of the food-handlers. The poor hygiene practice might have been confounded by the fact that most food-handlers were individuals from the lower socioeconomic class with low level of education. In addition, none of the food-handlers at the facilities had been appropriately trained in safe food-handling practices.

Although stages of intestinal parasites were not detected in the fingernails of food-handlers in the present study, earlier reports showed the presence of ova, larvae, and cysts of intestinal parasites under fingernails of study participants (12,22-24).

One limitation of the study was that, although all the food-handlers were included in the study, the sample size was small, and it was, thus, unable to use advanced analysis to make associations.

The prevalence of enteropathogens from stool specimens, fingernail contents, and intestinal parasites of the food-handlers was high in this study. An effective means of preventing the transmission of pathogens from food-handling personnel via food to consumers is strict adherence to good personal hygiene and to hygienic food-handling practices. It is recommended that education and training in good-hygiene practices should be provided to all food-handling personnel.

## ACKNOWLEDGEMENTS

The authors acknowledge the University of Gondar for financing the study. Their gratitude also goes to the study subjects for their participation in the study.
